# Role of community pharmacists in weight management: results of a national study in Lebanon

**DOI:** 10.1186/s12913-020-05258-7

**Published:** 2020-05-07

**Authors:** Mohamad Ali Hijazi, Hibeh Shatila, Abdalla El-Lakany, Hiba Al Rifai, Maha Aboul-Ela, Farah Naja

**Affiliations:** 1 Faculty of Pharmacy, Department of Pharmaceutical Sciences, Beirut Arab University, Beirut, Lebanon, P.O. Box: 11 5020, Beirut, Lebanon; 2 Nutrition and Food Sciences Department, Faculty of Agriculture and Food Sciences American, University of Beirut, Beirut, Lebanon

**Keywords:** Weight management, Obesity, Community pharmacy, Lebanon

## Abstract

**Background:**

Ideally situated within the community, pharmacists can be involved in a broad range of health promotion campaigns including prevention of obesity. Limited evidence is available regarding their involvement in weight management in Lebanon, a country with escalating prevalence rate of obesity.

**Objective:**

To examine the role of community pharmacists in weight management in Lebanon, specifically studying their beliefs, current practices, services, and knowledge.

**Methods:**

Using a stratified random sampling approach, a cross sectional national survey of community pharmacists was conducted (*n* = 341, response rate 89%). At the pharmacy, and through a face-to-face interview, pharmacists completed a multi-component questionnaire that addressed, in addition to socio-demographic and work characteristics, their beliefs, practices, knowledge in relation to weight management. Frequencies and proportions were used to describe the data. Simple and multiple linear regression analyses were used to examine the determinants of knowledge in the study population.

**Results:**

Over 80% of study participants agreed that they have an important role to play in weight management. However, 50% of pharmacists did not agree that weight loss products are well regulated and 81.1% thought that companies marketing weight loss products are making false promises. The majority of pharmacists always/often sold weight loss products (84.7%) and counseled their patients for diet (86.3%) and physical activity (91.7%). Despite taking weight and height measurements, 50% of pharmacists rarely/never calculated BMI. Among the pharmacists who reported side effects of weight loss products (46.5%), the majority (91.3%) did so to the pharmaceutical company. The knowledge of pharmacists was better for the use of weight loss products as opposed to their side effects and interactions. Significant predictors of knowledge were holding a Masters/ PhD degree in Pharmacy, graduating from a university inside Lebanon, obtaining weight management training within the academic degree, and receiving inquiries about weight management in the pharmacy more than once daily.

**Conclusions:**

The results of the study provided important insights on the beliefs, practices and knowledge of community pharmacists in weight management in Lebanon. These findings could be used to inform the development of future evidence-based community pharmacists led weight management service provision nationally and internationally.

## Background

Over the past 30 years, the prevalence of obesity has been increasing at an alarming rate worldwide. It is described as a global pandemic with currently more than 1.9 billion adults being overweight of which more than 650 million are obese [1]. Obesity is associated with increased morbidity and mortality and is a major risk factor in the etiology of many non-communicable diseases including type 2 diabetes, stroke, coronary artery disease, dyslipidemia, hypertension, pulmonary disease and cancers [2]. In addition to its physical consequences, obesity has also been linked to an increased risk for many psychiatric disorders, such as depression, impaired body image, low self-esteem, eating disorders, stress and poor quality of life [3]. The continuous rise in obesity is hence considered a global public health challenge and is threatening health improvements in many countries. Therefore, there is an eminent need to control the escalating burden of obesity through the implementation of evidence-based interventions for weight management [4].

Although physician recommendations have consistently been shown to exert a powerful influence on weight management, time constraints, limited access to resources for lifestyle changes, and low reimbursement are barriers between patient and provider [5]. Accumulating evidence suggested that a multidisciplinary approach that utilizes a broad range of health care expertise with proven synergy is most likely to be effective [6]. Among these health care expertise, community pharmacists are often considered the first contact point between patient and the health care system, perceived as accessible and trustworthy and have frequent contact with patients due to prescription dispensing schedules [7]. Ideally situated within the community, they can be involved in a broad range of health promotion campaigns and services, including prevention of chronic diseases such as hyperlipidemia, hypercholesterolemia, diabetes, osteoporosis, as well as obesity and weight management [8–13]. In fact, the American Society of Health-System Pharmacists recommends that pharmacists work with obese patients to manage lifestyle modifications [14]. A significant component of the pharmacists’ academic education covers etiology of obesity, its risk factors, management and treatment which qualifies as a strong foundation for providing lifestyle management counseling [15]. A recent scoping review of studies addressing the role of pharmacists in weight management counseling concluded that weight and obesity management interventions delivered by community pharmacies resulted in some weight loss, which, despite being modest, can be clinically significant as it improves surrogate markers of cardiovascular disease [16]. The majority of studies included in this scoping review were however conducted in developed countries [17–19] with limited evidence in many developing counties, including those in the Eastern Mediterranean Region (EMR), where the prevalence of obesity is escalating at an alarming rate [10, 20].

Lebanon, similar to neighboring countries in the EMR, is also witnessing a sharp increase in obesity prevalence with rates doubling between years 1997 and 2009. In 2009, among adults, obesity prevalence was 28.2% compared to 17.4% in 1997 [21]. This increase in obesity prevalence was also seen among children [21, 22]. The increase in obesity prevalence in Lebanon necessitates the inclusion of a multi-disciplinary team for a proper obesity management [23] among which pharmacists in Lebanon are highly trusted and recognized as having expertise in the health field [24]. In fact, a previous survey of community pharmacists in Lebanon investigating their role in managing hypertension showed that the majority advised and counseled patients on lifestyle habits and behaviors, addressing smoking, alcohol abuse, and healthy eating choices [24]. Furthermore, a recent study by our group examining the role of community pharmacists in Complementary and Alternative Medicine (CAM) showed that 80% of the surveyed pharmacists believed that providing information to customers about CAM products is a pharmacist’s professional responsibility, and 64.5% of pharmacists were always or often advising patients on safe use of CAM products and ask for their feedback after use [25]. These positive attitudes and practices of Lebanese pharmacists, in addition to their convenient and affordable accessibility, represent a potential opportunity to explore their role in weight management services. Therefore, the aim of this study is to examine the involvement of community pharmacists in Lebanon in overweight and obesity prevention and treatment, specifically studying their beliefs towards their role in weight management, current practices, services, and knowledge. In addition, the study aims to investigate the barriers to deliver optimal weight management services among community pharmacists in the country.

## Methods

In this study, a national cross-sectional survey of pharmacists in Lebanon was conducted between August 2018 and January 2019. The target population was pharmacists practicing in community pharmacies in Lebanon, while the study population included pharmacists recruited from a nationally representative sample of community pharmacies in the country. The sampling unit for this study was the pharmacy. A comprehensive list of community pharmacies in various governorates of Lebanon was obtained from the Order of Pharmacists in Lebanon (OPL). Pharmacies were chosen from this list using a stratified random sampling. The stratification was at the level of the six Lebanese governorates. Within each stratum (governorate), random samples of pharmacies were selected whereby numbers of pharmacies selected from each stratum was proportional to the total number of pharmacies in that particular stratum. In a few pharmacies, more than one pharmacists were present at the time of the conducting the survey, in which case one pharmacist was selected at random, using the Kish method [26]. Sample size calculations showed that a minimum of 342 pharmacists need to be recruited in order to detect an expected outcome at a prevalence of 50% with a 95% confidence interval (CI) and a margin of error of 5%. The calculations for sample size were conducted using Raosoft sample size calculator [27]. The study protocol was approved by the Institutional Review Board at the Beirut Arab University under the protocol number 2019H-0059-P-R-0300.

The study protocol involved conducting a survey among community pharmacists in the selected pharmacies. Data collection was carried out by field workers who were trained extensively on interviewing techniques and administration of the questionnaire. Prior to starting the survey, field workers introduced the study and assured the pharmacist of the confidentiality of data collected and that he/she has the right not to answer any of the questions in the questionnaire and to withdraw at any point during the survey. Interested pharmacists signed a written consent. Completing the survey lasted 20–25 min.

The development of the questionnaire used in the data collection for this study was designed by a detailed review of relevant past literature [15, 28–37]. The questionnaire was further reviewed by an expert panel of a community pharmacist, a professor of pharmacy, a nutrition epidemiologist, and a clinical nutritionist. The main aim of this review was to ensure content validity, clarity, as well as relevance to the Lebanese context. The questionnaire was written in English, translated to Arabic and then back translated to English to ensure parallel form reliability. The developed questionnaire was administered for pilot testing to 16 community pharmacists in various governorates in Lebanon. The pharmacies for the pilot test phase were conveniently selected. Results of the pilot test were not included in this study. The main changes that were introduced to the questionnaire, because of the pilot testing, were in relation to the technical translation of certain terms, in order to enhance the cultural and context specificity of the questionnaire. A copy of the English version of the questionnaire is found in Additional file 1. The final version of the questionnaire consisted of five sections: the first section addressed demographic characteristic of the pharmacist (age and gender), education information (level of education (Bachelors, Masters, Pharm D or PhD), location of the university he/she graduated from (inside or outside Lebanon), receiving education/training related to weight management during his/her university education years or post-graduation), employment (employment status, number of years of experience as community pharmacist), characteristic of the pharmacy (number of pharmacists in the pharmacy, duration for which the pharmacy open for) and frequency of queries/day the pharmacists receive about weight management products. The second section of the questionnaire focused on participants’ beliefs of a few issues related to weight management such as the role of the pharmacists in weight management, the exclusivity of sale of weight management product in pharmacies, whether or not they believe weight management should be addressed through a multidisciplinary team approach and whether other healthcare professions are more suited than pharmacists to deal with weight management and the need for continuous education for pharmacists in weight management. In addition, this section included the pharmacists’ perception regarding the regulatory framework of weight management products in Lebanon, the use/abuse of weight management products by consumers, and the role the companies and media play in orienting the consumers’ behavior. The third section of the questionnaire included questions assessing the pharmacist’s practices in weight management, such as selling, advising patient on safe use, reporting of adverse effects, checking for drug or food interactions, counseling patients on (behaviors related to weight management such as low calorie diets, physical activity, pharmacotherapy, etc.), the provision of weight related measurements (weight, height, waist circumference, Body Mass Index (BMI) calculations, blood glucose, body fat, blood pressure measurements), referrals of customers to dieticians and follow up on possible side effects of weight management. The fourth section of the questionnaire assessed pharmacist’s perceived barriers in providing weight management service such as time, staff, space, equipment, the need for additional payment, the lack of knowledge and interest. For sections 2, 3 and 4, responses were measured on a 5-point Likert scale (1-strongly agree, 2- agree, 3-neutral, 4-disgaree and 5-stronglt disagree). The last section of the questionnaire addressed the pharmacist’s knowledge toward weight management. Ten questions were selected to address general knowledge in overweight and obesity as well as the uses, side effects and drug interactions of commonly sold weight management products [15, 28–36]. The weight management products used in the questionnaire included herbal (green tea), laxative and orlistat; and were selected based on consultation with a panel of pharmacists who suggested that these products were the most commonly used products in the market for weight management in the Lebanese market. This panel of pharmacists consisted of the President of OPL, the chief executive officer of the largest wholesaler in Lebanon, and a sale manager from a pharmaceutical company. This panel, based on their experience and their sales data, suggested a list of commonly sold products. This list was further vetted by five community pharmacists, one from each of the Lebanese governorate, in order to select the three most commonly sold products for weight management in the country.

A copy of the questionnaire used in data collection is provided as supplementary file to this manuscript.

### Statistical analysis

Data collected were entered into the Statistical Package for Social Sciences (SPSS, version 25). Accuracy of the collected data was evaluated by visual inspection and examination of ranges and logic checks. Frequencies and proportions were used to summarize the subjects’ characteristics, as well as the beliefs, practices, barriers and knowledge of study participants. For knowledge, in addition to the aforementioned descriptive statistics, a knowledge score corresponding to the sum of points a pharmacist obtains for all the knowledge questions, whereby he/she is given one point for a question that they answered correctly and ‘0’ point if their answer was either wrong or ‘I don’t know’ answer’. Therefore, the knowledge score ranged between ‘0’ and ‘10’, with higher values of the score reflecting more correct answers and hence better knowledge. In order to determine the correlated of knowledge, simple and multiple linear regression analyses were conducted. In these regression analyses, the knowledge score was considered as the outcome variable while the socio demographic factors were entered as independent variables. Of the socio-demographic characteristics, variables that were found significantly associated with knowledge score in the simple regression were entered in the multiple regression. Regression diagnostics were examined to ensure that the assumptions for the MLR were met, including linearity between the independent and the dependent variables, homoscedasticity, and multicollinearity. The results of the diagnostics are presented in Additional file 2. A *p*-value < 0.05 was considered statistically significant.

## Results

Three hundred and eighty three pharmacists were approached, 341 of whom agreed to participate in the study (89% response rate). The distribution of the pharmacies in Lebanon and the study sample, across the 6 Lebanese governorates are shown in Table 1. The study sample showed similar proportions of pharmacies among the various governorates.

**Table 1:** Distribution of pharmacies across governorates in this study in comparison to national distribution of pharmacies *n* = 341

	Pharmacies in the study *n*(%)	Pharmacies in Lebanon *n*(%)
**Beirut**	27(7.9)	238(7.8)
**South**	40(11.7)	353(11.6)
**North**	50(14.7)	436(14.3)
**Mount Lebanon**	147(43.1)	1311(43.1)
**Beqaa**	51(15.0)	482(15.8)
**Nabatieh**	26(7.6)	223(7.3)
**Total**	**341**	**3043**

The characteristics of the study population are presented in Table 2.The pharmacists were of varied age groups, with most of them aged 40 and below (66.4%). The proportion of males (51.9%) and females (48.1%) were comparable. The study sample included pharmacists who were employed in the selected pharmacies (41.8%) or owners of these pharmacies (58.2%). As for the education level, more than half of the study population were holders of a Bachelor’s degree (57.5%), while 42.6% had attained higher degrees: 22.3% a Pharm D degree and 20.2% a Masters or PhD degree. Sixty-four percent of the pharmacists attained their university education in Lebanon. Only 35.3% of the pharmacists reported receiving weight management training during their university education and a lower proportion (21.5%) underwent a post-graduation training in weight management. Forty-four percent of the pharmacists had more than 10 years of work experience, followed by 32.1% having 4–10 years of experience and 24.1% having 1-3 years of experience. Most of the pharmacists work in a pharmacy that has been open for more than three years (81.1%). Almost half of the pharmacists (47.5%) reported receiving more than 1 query a day about weight management products.

**Table 2:** Characteristics of study sample. (*n* = 341)

	Frequency	Percentage
**Age range**		
< 30 years	114	33.5
31–40 years	112	32.9
≥ 41 years	114	33.5
**Gender**		
Male	177	51.9
Female	164	48.1
**Employments status**		
Employed	142	41.8
Pharmacy owner	198	58.2
**Highest educational level attained**		
Bachelors	196	57.5
Pharm D	76	22.3
Masters/PhD	69	20.2
**University you graduated from**		
Outside Lebanon	123	36.3
In Lebanon	216	63.7
**During your university education, did you receive any weight management training?**		
Yes	120	35.3
No	220	64.7
**Did you receive any postgraduate education/training on weight management?**		
Yes	73	21.5
No	266	78.5
**Years of work experience**		
1–3 years	82	24.1
4–10 years	109	32.1
Above 10 years	149	43.8
**How many pharmacists work in this pharmacy?**		
1–2 pharmacist	263	77.4
3–5 pharmacist	74	21.8
More than 5 pharmacist	3	0.9
**How long has this pharmacy been opened for?**		
1–3 years	64	18.9
4–10 years	117	34.5
Above 10 years	158	46.6
**How many times per day do you get queries about weight management products at your pharmacy?**		
Once daily or less	179	52.5
More than once daily	162	47.5

The majority of pharmacists (87.9%) were strongly agreeing or agreeing that obesity is a growing problem in Lebanon. Overall, the study participants strongly agreed or agreed that pharmacists have a role to play in the field of weight management (84.8%), that providing information about weight management products is a pharmacist’s professional responsibility as extension of their role as health professional (83.1%) and that weight management products should be sold only in pharmacies (83.8%) (Table 3). A total of 82.7% of participants strongly agreed or agreed that a multidisciplinary team approach to weight management will work best (82.7%). Eighty-two percent strongly agreed or agreed that continuous education of pharmacist should include weight management and training. In general, the participant showed negative beliefs toward the market by strongly agreeing or agreeing that customers are abusing weight management drugs (76.9%) and companies are abusing customers by making false promises (81.1%). In addition, more than half (54.9%) disagreed or strongly disagreed that that weight loss products are well regulated.

**Table 3:** Community pharmacists’ beliefs towards their role in weight management^*^. (*n* = 341)

	Strongly agree	Agree	Neutral	Disagree	Strongly disagree
Do you think obesity is a growing problem in Lebanon	221(66.8)	70(21.1)	27(8.2)	9(2.7)	4(1.2)
Do you believe that pharmacists have a role to play in the field of weight management	198(58.1)	91(26.7)	24(7.0)	17(5.0)	11(3.2)
Providing information about diet products is a pharmacist’s professional responsibility as extension of their role as health professional	166(49.4)	114(33.7)	35(10.4)	14(4.1)	9(2.7)
Do you think that weight loss products should be sold only in pharmacies	235(69.3)	49(14.5)	17(5.0)	22(6.5)	16(4.7)
Do you feel other healthcare professionals are more appropriately suited to be involved in this area	135(40.1)	105(31.2)	60(17.8)	26(7.7)	11(3.3)
Do you think that multidisciplinary team approach to weight management will work the best	196(58.8)	80(23.9)	34(10.1)	16(4.8)	9(2.7)
Continuous education of the pharmacist should include weight management and training	180(53.3)	97(28.7)	40(11.8)	12(3.6)	9(2.7)
Do you think that customers are abusing weight loss products	163(48.8)	94(28.1)	46(13.8)	24(7.2)	7(2.1)
Do you think that companies marketing weight loss products are making false promises?	174(52.3)	96(28.8)	32(9.6)	19(5.7)	12(3.6)
Do you believe that herbal weight loss products are well regulated?	37(10.9)	53(15.6)	63(18.6)	79(23.3)	107(31.6)
Do you think that media and advertisements are playing a positive role in educating customers towards weight loss products and weight management	112(33.0)	54(15.9)	43(12.7)	52(15.3)	78(23.0)

^*^Values in this table represent *n* (%)

The majority of the pharmacists (84.7%) participating in this study reported that they always/often sell weight loss products in their pharmacy and 88.7% reported always/often being asked for weight loss products (Table 4). Eighty-six percent reported always/often counselling customers who request to buy products for weigh management on the safe and effective use of the product and 77.6% always/often check for drug or food interaction while dispensing weight loss products. The majority were always/often advising the patients to eat low calorie diet (86.3%), increase physical activity (91.7%), and increase consumption of soluble fibers (80.9%). In addition, participated pharmacists reported that they always/often provide weight and height measurement for their patients (80 and 60%, respectively). However, the majority (81.7%) were rarely/never providing waist circumference measurements for patients. Most participants answered always/often provide blood glucose and blood pressure measurements (83.7 and 89.0%, respectively) at their pharmacy, but only 15.3% of them provided body fat measurement. Around 66% of the surveyed pharmacists reported referring patients to dieticians, on as needed basis. Most of the pharmacists (73.6%) reported that they always/often ask customers for any side effects or undesirable reactions after taking weight loss products; however, 53.5% of the pharmacists rarely/never reported any toxicity or adverse reaction of weight loss products. Among those who reported the incidence of toxicity or adverse reactions, 91.3% of pharmacists were reporting to the pharmaceutical companies and only 1.9% reported to the OPL, whereas the remaining reported to physician (3.9%) and MOPH (2.9%) (Table 5).

**Table 4:** Current practice towards weight management services among community pharmacists in Lebanon^*^. (*n* = 341)

	Always	Often	Sometimes	Rarely	No
Do you dispense weight loss products at your pharmacy	188(55.5)	99(29.2)	32(9.4)	14(4.1)	6(1.8)
Do your patients ask you for weight loss products	212(62.7)	88(26.0)	21(6.2)	8(2.4)	9(2.7)
Do you counsel customer who request to buy products for weight management on the safe and effective use of the product	224(66.9)	65(19.4)	26(7.8)	10(3.0)	10(3.0)
Do you check for drug or food interaction while dispensing weight loss product	193(57.8)	66(19.8)	35(10.5)	22(6.6)	18(5.4)
Do you advice the patients to eat low calorie diet	235(69.7)	56(16.6)	30(8.9)	8(2.4)	8(2.4)
Do you advice the patients to increase physical activity	262(78.0)	46(13.7)	14(4.2)	8(2.4)	6(1.8)
Do you advice the patients to increase consumption of soluble fiber	214(63.9)	57(17.0)	35(10.4)	19(5.7)	10(3.0)
Do you provide weight measurements for patients	222(66.1)	46(13.7)	22(6.5)	14(4.2)	32(9.5)
Do you provide height measurements for patients	150(44.6)	45(13.4)	18(5.4)	23(6.8)	100(29.8)
Do you provide waist circumference measurements for patients	21(6.3)	15(4.5)	25(7.5)	34(10.1)	240(71.6)
Do you provide BMI calculation for your patients	101(30.0)	33(9.8)	38(11.3)	28(8.3)	137(40.7)
Do you provide blood glucose measurement at your pharmacy	243(71.9)	40(11.8)	16(4.7)	10(3.0)	29(8.6)
Do you provide blood pressure measurement at your pharmacy	253(75.1)	47(13.9)	13(3.9)	3(0.9)	21(6.2)
Do you provide body fat measurement at your pharmacy	39(11.5)	13(3.8)	21(6.2)	15(4.4)	250(74.0)
Do you refer your patient to dieticians when needed	156(46.3)	69(20.5)	58(17.2)	17(5.0)	37(11.0)
Do you ask customers for any side effect or undesirable reaction after taking weight loss products	186(55.2)	62(18.4)	44(13.1)	11(33.3)	34(10.1)
Do you report any toxicity or adverse reaction of weight loss products?	89(26.5)	31(9.2)	36(10.7)	27(8.0)	153(45.5)

^*^Values in this table represent *n* (%)

**Table 5:** To whom do you report any toxic or undesirable effect that occurred with patients using weight loss products??

	*n* = 103	%
**Manufacturing company**	94	91.3
**Ministry of Public Health (MoPH)**	3	2.9
**Order of Pharmacy in Lebanon (OPL)**	2	1.9
**Physician**	4	3.9

Over 30% strongly agreed/agreed to the following barriers to weight management services; I don’t have time (30.4%), I don’t have enough staff (36.5%), I don’t have enough space to have private consultation area (37%), I don’t have the relevant equipment (39.8%), I would need additional payment (31.8%). On the other hand, fewer pharmacists strongly agreed or agreed that adequate knowledge (21.5%) or interest in the weight management (18.6%) were barriers for them to provide weight management services (Table 6).

**Table 6:** Barriers in providing weight management services among community pharmacists in Lebanon.^*^ (*n* = 341)

	n(%)				
	Strongly agree	Agree	Neutral	Disagree	Strongly disagree
I don’t have enough time to provide weight management services	55(16.2)	48(14.2)	59(17.4)	64(18.9)	113(33.1)
I don’t have enough staff to provide weight management services	70(20.6)	54(15.9)	51(15.0)	66(19.5)	98(28.9)
I don’t have enough space to have a private consultation area to provide weight management services	74(21.9)	51(15.1)	39(11.5)	59(17.5)	115(34.0)
I don’t have the relevant equipment (e.g. weighing scale,etc) to provide weight management services	78(23.0)	57(16.8)	41(12.1)	56(16.5)	107(31.6)
I would need additional payment to provide weight management services	69(20.4)	38(11.2)	40(11.8)	43(12.7)	148(43.8)
I don’t have the enough knowledge to provide weight management services	36(10.6)	37(10.9)	47(13.9)	72(21.2)	147(43.4)
I don’t have the interest to provide weight management services	36(10.6)	27(8.0)	48(14.2)	76(22.4)	152(44.8)

^*^Values in this table represent *n* (%)

Table 7 displayed the results of self-knowledge assessment of community pharmacists with regards to weight management. The assessment included 10 questions addressing general knowledge about overweight and obesity as well as the uses, side effects and interactions of commonly sold weight management products in Lebanon, namely green tea, herbal laxative and orlistat. While 71.1% of pharmacists answered correctly the question related to BMI cutoff for obesity, only 25% knew about weight loss targets and 51% realized the importance of weight maintenance, after reaching the target weight. The percentage of pharmacists answered correctly the side effects and interactions of laxatives was greater than those for green tea and olistat (83.4 and 79.2% for laxatives as compared to 45.2 and 17.3% for green tea and 67.1 and 54.6% for olistat).

**Table 7:** Evaluation of self-knowledge towards weight management among community pharmacists in Lebanon. (*n* = 341)

	True/ False	% answered correctly	% answered incorrect	% answered I don’t know
The cut off for body mass index (BMI) to indicate obesity is > 29.9 kg/m^2^.	T^15^	239(71.1)	41(12.2)	56(16.7)
An initial weight loss goal should be to lose more than 10% of current body weight in 6 months	F ^27,28,36^	82(24.3)	175(51.9)	80(23.7)
Once a weight loss goal is achieved it is okay to discontinue treatment.^a^	F^29^	172(51.0)	146(43.3)	19(5.6)
Laxatives are considered very useful method to lose weight in obese persons.	F^30^	262(77.5)	66(19.5)	10(3.0)
Herbal Laxative (like senna, cascara, etc) are recommended for pregnant or breast feeding women	F^31^	282(83.4)	42(12.4)	14(4.1)
High consumption of green tea may exert toxicity to liver cells.	T^32^	152(45.2)	71(21.1)	113(33.6)
Orlistat use is associated with a higher incidence of gastrointestinal adverse events compared with placebo.	T^33^	226(67.1)	71(21.1)	40(11.9)
The chronic use laxatives may potentiate the effects of diuretics which may lead to significant losses of fluid and electrolytes, including sodium, potassium, magnesium and zinc.	T^34^	267(79.2)	41(12.2)	29(8.6)
Concomitant use of large quantities of green tea may increase the effectiveness of anticoagulation drugs (warfarin).	F ^30,31,35^	58(17.3)	143(42.7)	134(40.0)
Orlistat is contraindicated in patients with cardiovascular diseases.	F^33^	184(54.6)	109(32.3)	44(13.1)

^a^ Interviewers were trained to indicate that ‘treatment’ in this question referred to ‘dietary changes’

For the overall score of knowledge, results of the multiple linear regression revealed few factors that were associated with a higher score, indicated by a significantly positive value for ß. The magnitude of the latter indicated the difference in knowledge score between the categories compared. These factors included: Holding a Masters or PhD degree in Pharmacy as compared to Bachelor’s degree (ß =0.79, 95%CI: 0.37–1.21), graduating with a pharmacy degree from a university in Lebanon as opposed to abroad (ß =0.88, 95%CI: 0.5–1.26), obtaining weight management training within the academic degree (ß =0.42, 95%CI: 0.08–0.75), and receiving inquiries about weight management in the pharmacy more than once daily (ß =0.41, 95%CI: 0.1–0.73) (Table 8).

**Table 8:** Predictors knowledge score (related to weight management products) among community pharmacists in Lebanon. *n* = 341

	B, 95% CI	Adjusted B, 95% CI
**Age range**		
< 30 years	Ref	Ref
31–40 years	−0.32(−0.72, 0.09)	0.05 (−0.44,0.54)
≥ 41 years	**−0.73(−1.13, − 0.32)**	−0.10(− 0.75,0.56)
**Gender**		
Male	Ref	Ref
Female	**0.37 (0.04,0.71)**	0.17 (−0.17,0.51)
**Employments status**		
Employed	Ref	–
Pharmacy owner	0.18(−0.17, 0.52)	–
**Highest educational level attained**		
Bachelors	Ref	Ref
Pharm D	0.26(−0.16,0.68)	0.13(−0.27,0.53)
Masters /PhD	**0.61 (0.19,1.04)**	**0.79(0.37,1.21)**
**Which university did you graduate from**		
Outside Lebanon	Ref	Ref
In Lebanon	**0.88(0.55,1.22)**	**0.88(0.50,1.26)**
**During your university education, did you receive any weight management training?**		
No	Ref	Ref
Yes	**0.45(0.10, 0.80)**	**0.42(0.08,0.75)**
**Did you receive any postgraduate education/training on weight management?**		
No	Ref	–
Yes	0.14(−0.27,0.55)	–
**Years of work experience**		
1–3 years	Ref	Ref
4–10 years	−0.16(− 0.62,0.29)	−0.15(− 0.63,0.34)
Above 10 years	**−0.52(− 0.94,-0.10)**	−0.16(− 0.80,0.48)
**How many pharmacists work in this pharmacy?**		
1–2 pharmacist	Ref	–
3–5 pharmacist	0.12(−0.30, 0.53)	–
More than 5 pharmacist	−0.71(−2.88, 1.46)	–
**How long has this pharmacy been opened for?**		
1–3 years	Ref	–
4–10 years	−0.25 (− 0.74, 0.23)	–
Above 10 years	− 0.46(− 0.93,0.02)	–
**How many times do you get queries about weight management products at your pharmacy?**		
Once daily or less	Ref	Ref
More than once daily	**0.42(0.09, 0.76)**	**0.41(0.10, 0.73)**

## Discussion

This is the first national study to examine the role of pharmacists in weight management in Lebanon and to shed light on their beliefs, practices and knowledge regarding weight management and weight loss products. Overall, the study findings highlighted a general positive belief among pharmacists regarding their role in weight management while acknowledging the role of other health care professionals in managing overweight and obesity. Pharmacists participating in this study, however, expressed reservations towards the regulatory framework governing the market of weight loss products in the country. With regards to practices, despite engaging in counseling for weight management as well as dispensing weight loss products, a sizeable proportion of pharmacists were rarely carrying out important diagnostic measures, such as BMI calculations or waist circumference measurements. In addition, even though 73% of pharmacists in this study inquired about products’ side effects, only 35% reported such effects with the majority doing so to the company selling the products or to its medical representative. This study revealed important gaps in the knowledge of pharmacists, most importantly with regards to the side effects of certain weight loss products and their interaction with other medications. In this study, factors significantly associated with better knowledge were higher education (Masters or PhD), earning the pharmacy degree from a university in Lebanon, obtaining formal education in weight management, and receiving frequent inquiries about weight management from patients/customers.

In this study, the majority of pharmacists believed that their role is important in weight management and that dispensing diet and weight loss products ought to be the pharmacist responsibility solely. More specifically, the findings indicated that while pharmacist perceived that they have a role to play in the area of weight management, they also believed in the importance and effectiveness of a multidisciplinary team and that other health care professionals may be better suited to take lead on that front. These findings are in line with previous research, whereby a survey of 128 community pharmacists in Scotland showed positive attitudes to the provision of healthy weight management services [38]. Furthermore, a qualitative study in Australia investigating the pharmacists’ opinion about provision of weight management services indicated that participants clearly perceived an important role for pharmacists in weight management, as part of a multi-disciplinary team [17]. Also in Australia, a more recent study conducted among pharmacy students and early career pharmacists showed that participants were positive about their perceived role in providing weight management services [39].

These positive beliefs of pharmacists towards their role in weight management were contrasted with the doubts that many surveyed pharmacists casted on the regulations governing the market for CAM products including those for weight loss. Such doubts were also reported by other studies [25, 40–42]. In fact, since only few of the hundreds products sold in pharmacies for weight loss are licensed medicines, they are not subject to the strict control required for medicines, in terms of efficacy, safety, quality or provision of a standardized patient information leaflet [43]. In Lebanon, several weight loss products were promoted and sold for many years before the Ministry of Public Health (MoPH) withdrew them from the market and prevented their uses because of contamination, presence of unauthorized ingredients, or counterfeits [44].

In this study, the positive belief of the surveyed pharmacists regarding their role in weight management was also reflected in their practices, whereby the majority of pharmacists always/often dispensed weight loss products and counseled patients on healthy lifestyle habits, including diet and physical activity. A few studies reported more elaborate interventions for weight loss as part of the pharmacist’s role such as patient-centered weight loss programs [45, 46] and conducting slimming courses [47]. However, and in support of the study findings, the supply of medication and counseling for weight loss remain the two most common pillars of the pharmacist role in weight management [17]. Alarmingly, in this study, a few practices that were reported by the surveyed participants jeopardized the efficacy and safety of their role in weight management. For instance, although the pharmacists reported taking height and weight measurements, only a few calculated the BMI or provided assessments for waist circumference and body fat, raising questions regarding the sensitivity of their diagnosis of obesity and consequently the relevance of their consultation. It is indisputable that weight and height measurements alone are poor indicators of obesity, if not used to calculate BMI. In fact, most of the scientific reports call for other anthropometric measurements (in addition to height and weight) such as waist circumference and body fat to classify obesity [48].

Another disconcerting finding in this study is related to the practices of dispensing weight loss medications. First, over 40% of surveyed pharmacists did not discuss with their patients the side effects or undesirable reactions of these medications. Certain drugs advertised for weight loss could pose significant adverse effects, for example *Garcinia cambogia* extract and hepatic failure [49] and low dose Human Chorionic Gonadotrophin injections and increased thrombosis risk [50]. According to the American Pharmacists Association, a patient-centered discussion on safe and effective medication use is among the main medication-related responsibilities of pharmacists as it is directly linked to improving patient safety [51]. Therefore, it is important for the pharmacist to heighten the patients’ awareness of potential health implications while dispensing weight loss medication. Second, over 50% of surveyed pharmacists indicated that they do not report toxicity or adverse effects of drugs when encountered. Moreover, of the few pharmacists who reported these adverse effects, the vast majority did so to the manufacturing/distributing company. Similar practices were also observed among community pharmacists in Lebanon in the case of complementary and alternative products [25]. These practices threaten public health safety, since many of the adverse effects remain not reported and could lead to serious health implications. Furthermore, communicating these adverse effects to the manufacturing/distributing company, whose main objective is financial gain from drug sales, raises a serious ethical question over such practices. Alternatively, it could be argued that such a practice stemmed from the fact that a large proportion of pharmacists reported little trust in the regulatory bodies of the market in Lebanon. In both cases, these findings call upon the MoPH to upscale the health care system and implement a proper reporting system for the pharmacists and other healthcare provider as well.

In this study, the beliefs and practices of pharmacists with regards to weight management were contrasted by significant knowledge gaps in this domain. More specifically, over 50% of participants in this study missed critical concepts such as the recommended percent weight to be lost over a certain period of time and the need to maintain healthy dietary habits after the target weight is reached. Furthermore, despite the fact that the majority of pharmacists in this study were involved in dispensing weight loss products, a sizeable proportion answered incorrectly the questions related to side effects and interactions of these products. Such knowledge gaps were also identified in previous studies, whereby pharmacists indicated the need for trainings in weight loss consultations, overweight and obesity diagnosis and uses of weight loss products (including side effects and interactions with other drugs) [38, 39].

An important factor was found to be associated with better knowledge, was receiving formal education in weight management. In Lebanon, although the academic curricula of pharmacy education have been evolving to match the shift in pharmacy profession from solely dispensing medicine towards a more patient centered delivery of healthcare services, more focus on weight management services is still needed. In fact, many authorities in the field of pharmacy education, including the International Pharmaceutical Federation (FIP), and the Accreditation Council for Pharmacy Education (ACPE), called for reforms in pharmacy education to include courses on nutrition and lifestyle counselling. The implementation of these reforms was shown to improve knowledge about many aspects of weight management [52–54]. Related to pharmacy education, the results of this study showed that attaining a Masters or PhD of pharmacy was found to positively influence knowledge of weight management. This finding further underscored the role of formal education in enhancing the foundational knowledge in health and wellness. Another factor found to be associated with knowledge was obtaining the degree of pharmacy from a university inside Lebanon. A potential explanation of this association is that pharmacists studying in the country may be more literate in the health and social constructs of the society than those who studied abroad.

Examining the barriers that pharmacists faced in providing weight management services was essential to provide a more comprehensive situation analysis. The results of this study showed that considerable proportions (more than 30%) of surveyed pharmacists agreed that resources in terms of time, space, staff, remuneration, and relevant equipment were all common barriers to providing weight management services. The majority of these barriers were also cited in previous studies [10, 15, 17, 38]. A recent study showed that the pharmacy profession in Lebanon has been facing multiple challenges relating to the practicing of the profession and the protection of the professional status of pharmacists. More specifically pharmacists in Lebanon were found to be dissatisfied with multiple issues including the distribution of pharmacies, drug prices, profit margin, low income, workload, policies governing the profession, prescribing ethics, sale of counterfeit drugs and political intervention [55]. Recommendations proposed to overcome the ‘time’ barrier included (in addition to proper staff) the formation of multi-disciplinary practice models, within which pharmacists and other health care team members work together [15] and increasing the involvement of technicians and students/residents in the dispensing role, hence freeing the pharmacist for counseling [56]. Regarding space limitations, it is argued that the physical space in the majority of pharmacies is designed to support the drug dispensing role of the pharmacist with little room for counseling. For the latter, space is needed not only to provide the physical setting but also to ensure confidentiality of personal information often shared during weight management counseling. In Lebanon, such a barrier still existed despite that, according to OPL Decree No. 2622 issued in 1992, a section in the pharmacy should be dedicated to reception and counseling of patients. In fact, this decree specified that a minimum of 32 m^2^ is required to register a pharmacy which should be divided into four sections:1-reception of customers, 2-department of medicine cabinets, 3-laboratory dedicated to the preparation of prescriptions, 4 -warehouse of goods and medicines [57]. A few studies attempted at altering the lay out of the pharmacy placing the pharmacist in front of the counter, rather than behind. The results of these studies showed promising results in allowing a more prominent counseling role of the pharmacist [58, 59].

This is the first study to examine the role of pharmacists in weight management in Lebanon. The strengths of this study included the recruitment of a nationally representative sample of community pharmacies. The high response rate further enhances the external validity of the study findings. To achieve such a response rate, the field workers were trained to present the objectives of the research in a manner to illicit the interest of the pharmacist and hence increase his/her chance of participation**.** Despite this high response rate, 42 pharmacists who were approached did not agree to participate in this study. Therefore, a non-respondent bias could have affected our results. However, the likelihood that pharmacists who were less interested in weight management were more likely to decline participation, further underscores the implications of this study’s findings. In addition, by addressing various aspects such as beliefs, practices, knowledge and barriers, the study provided a comprehensive assessment allowing for the development of evidence based interventions to enhance the role of pharmacists in weight management. The data collection was conducted using an interview-based survey technique, therefore limiting reporting errors and decreasing missing answers. It is important to note, however, that the results of the study ought to be considered in light of a few limitations. First, the cross sectional design of the survey prevented any inference about causality. The direct association observed between the frequency of queries regarding weight management and knowledge could be an example of reverse causality precipitated by the design of the survey. Second, although field workers were extensively trained on maintaining a neutral attitude and on non-judgmental reaction, the possibility of a social desirability bias could not be ruled out and the results of the study could have been skewed in a manner to satisfy the person administering the questionnaire. That said, it is important to note that despite this potential bias, certain mal practices were observed at high rates. Alternative methods to conducting the survey, such as mailing or emailing the questionnaires, were not possible in the context of the study, given that not all pharmacists possess a mailing address or have access to emails.

## Conclusions

The results of this study showed that community pharmacists in Lebanon have a general positive belief regarding their role in weight management and are engaged in counseling for weight loss and dispensing weight loss products. In addition, the study findings highlighted important gaps in current practices of the pharmacists (such as failing to report side effects of drugs) as well as in their knowledge, specifically with regards to side effects of certain products and their interaction with other medications. In this study, higher education (Masters or PhD), earning the pharmacy degree from a university in Lebanon, obtaining formal education in weight management, and receiving frequent inquiries about weight management from patients/customers were all significant predictors of better knowledge. In addition, time, space, staff, remuneration, and relevant equipment were all common barriers to providing weight management services among community pharmacists in Lebanon. In conclusion, the findings of the study provided important insights on the beliefs, practices and knowledge of community pharmacists in weight management in Lebanon. Concerted efforts of multiple stakeholders including the Ministry of Public Health, Ministry of Education together with the OPL, are needed to promote the active role of community pharmacists in order to better address the escalating rates of obesity in the country. The findings of this study could be used to inform the development and provision of future evidence based community pharmacists led weight management service nationally and internationally.

## Supplementary information

**Additional file 1.**

**Additional file 2.**

## Data Availability

The survey questionnaire is included as Additional file. The datasets used and analyzed during the current study are available from the corresponding author on reasonable request.

## Abbreviations

BMI: Body Mass Index
OPL: Order of Pharmacists in Lebanon
CI: Confidence interval
FIP: International Pharmaceutical Federation
ACPE: Accreditation Council for Pharmacy Education
