# Supplementary material for: Role of community pharmacists in weight management: results of a national study in Lebanon
Source: BMC Health Serv Res. 2020 May 7;20:386. doi: 10.1186/s12913-020-05258-7 (PMC7204056; doi:10.1186/s12913-020-05258-7)
Supplement: Supplementary file 1 — Additional file 1. [file 12913_2020_5258_MOESM1_ESM.docx]

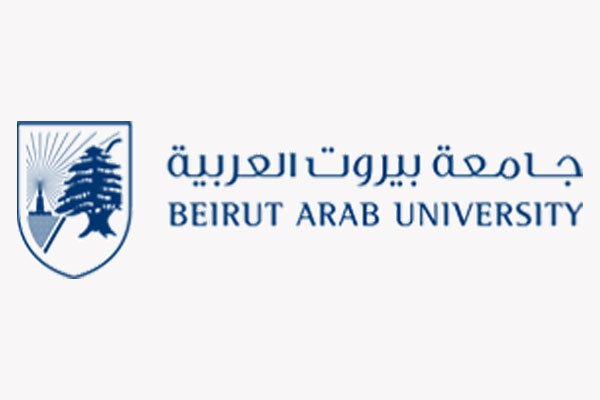


Role of community pharmacists in weight management: A national study in Lebanon

Date (dd /mm/ yy): ___/___/___

Subject ID: ______________________

Interview date:

Interview time:

Interviewer name:

District of the Pharmacy:

- Beirut
- South
- North
- Mount Lebanon
- Beqaa
- Nabatieh

**Section A: Socio-demographics**

Mark with an (X) for the suitable answer:

- Age range:
- < 30 years
- 31 – 40 years
- ≥ 41 years
- Gender:
- Male
- Female
- Employment status
- Full-time
- Part-time
- Pharmacy owner
- Highest educational level attained:
- Bachelors
- Masters
- Pharm D
- Ph.D.

Do you have any additional certificates? Please specify ---------------------------------

- Which university did you graduate from:
- LAU
- USJ
- BAU
- LU
- LIU
- Outside Lebanon
- During your university education, did you receive any Weight Management (WM) training?
- Yes
- No
- Did you receive any postgraduate education/training on weight management?
- Yes
- No
- Years of work experience:
- 1 – 3 years
- 4 – 10 years
- Above 10 years
- How many pharmacists work in this pharmacy?
- 1 – 2 pharmacists
- 3 – 5 pharmacists
- More than 5 pharmacist
- How long has this pharmacy been opened for?
- 1 – 3 years
- 4 – 10 years
- Above 10 years
- How many times do you get queries about weight management products at your pharmacy:

--------------------- times per day

--------------------- times per week

--------------------- times per month

**Section B: Pharmacist beliefs towards their role in weight management**

| **Statement** | **1** | **2** | **3** | **4** | **5** |
| --- | --- | --- | --- | --- | --- |
| ***Scale of 1-5 (1 = strongly agree, 2= agree, 3 = neutral, 4 = disagree, 5 = strongly disagree**) | | | | | |
| Do you think obesity is a growing problem in Lebanon |  |  |  |  |  |
| Do you believe that pharmacists have a role to play in the field of weight management |  |  |  |  |  |
| Providing information about diet products is a pharmacist’s professional responsibility as extension of their role as health professional |  |  |  |  |  |
| Do you think that weight loss products should be sold only in pharmacies |  |  |  |  |  |
| Do you feel other healthcare professionals are more appropriately suited to be involved in this area |  |  |  |  |  |
| Do you think that multidisciplinary team approach to weight management will work best |  |  |  |  |  |
| Continuous education of the pharmacist should include weight management and training |  |  |  |  |  |
| Do you think that customers are abusing weight loss products? |  |  |  |  |  |
| Do you think that companies marketing weight loss products are making false promises? |  |  |  |  |  |
| Do you believe that herbal weight loss products are well regulated? |  |  |  |  |  |
| Do you think that media and advertisements are playing a positive role in educating customers towards weight loss products and weight management |  |  |  |  |  |

**Section C: Current practice towards weight management services**

| **Statement** | **1** | **2** | **3** | **4** | | **5** | |
| --- | --- | --- | --- | --- | --- | --- | --- |
| ***Scale of 1-5: (1 (always), 2 (often), 3 (sometimes), 4 (rarely), and 5 (no)** | | | | | | | |
| Do you dispense weight loss products at your pharmacy? |  |  |  | |  | |  |
| Do your patients ask you for weight loss products? |  |  |  | |  | |  |
| Do you counsel customer who request to buy products for weight management on the safe and effective use of the product? |  |  |  | |  | |  |
| Do you check for drug or food interaction while dispensing weight loss product? |  |  |  | |  | |  |
| Do you advice the patients to eat low calorie diet? |  |  |  | |  | |  |
| Do you advice the patients to increase physical activity? |  |  |  | |  | |  |
| Do you advice the patients to increase consumption of soluble fiber? |  |  |  | |  | |  |
| Do you provide weight measurements for patients? |  |  |  | |  | |  |
| Do you provide height measurements for patients? |  |  |  | |  | |  |
| Do you provide waist circumference measurements for patients? |  |  |  | |  | |  |
| Do you provide BMI calculation for your patients? |  |  |  | |  | |  |
| Do you provide blood glucose measurement at your pharmacy? |  |  |  | |  | |  |
| Do you provide blood pressure measurement at your pharmacy? |  |  |  | |  | |  |
| Do you provide body fat measurement at your pharmacy? |  |  |  | |  | |  |
| Do you refer your patient to dieticians when needed? |  |  |  | |  | |  |
| Do you ask customers for any side effect or undesirable reaction after taking weight loss products? |  |  |  | |  | |  |
| Do you report any toxicity or adverse reaction of weight loss products?  If yes, to whom--------------------------------------------------------- |  |  |  | |  | |  |

**Section D: Pharmacist barriers in providing weight management** **services** (WMS)

| **Statement** | **1** | **2** | **3** | **4** | **5** |
| --- | --- | --- | --- | --- | --- |
| ***Scale of 1-5 (1 = strongly agree, 2= agree, 3 = neutral, 4 = disagree, 5 = strongly disagree**) | | | | | |
|  |  |  |  |  |  |
| I don’t have enough time to provide weight management services |  |  |  |  |  |
| I don’t have enough staff to provide weight management services |  |  |  |  |  |
| I don’t have enough space to have a private consultation area to provide weight management services |  |  |  |  |  |
| I don’t have the relevant equipment (e.g. weighing scale,etc) to provide weight management services |  |  |  |  |  |
| I would need additional payment to provide weight management services |  |  |  |  |  |
| I don’t have the enough knowledge to provide weight management services |  |  |  |  |  |
| I don’t have the interest to provide weight management services |  |  |  |  |  |

**Sector E: Evaluation of self-knowledge towards weight management**

| **Statement** | **True** | **False** | **I don’t know** |
| --- | --- | --- | --- |
| The cut off for body mass index (BMI) to indicate obesity  is > 29.9 kg/m^2^ |  |  |  |
| An initial weight loss goal should be to lose more than 10% of current body weight in 6 months |  |  |  |
| Once a weight loss goal is achieved it is okay to discontinue treatment |  |  |  |
| Laxatives are considered very useful method to lose weight in obese persons |  |  |  |
| Herbal Laxative (like senna, cascara, etc) are recommended for pregnant or breast feeding women |  |  |  |
| High consumption of green tea may exert toxicity to liver cells |  |  |  |
| Orlistat use is associated with a higher incidence  of gastrointestinal adverse events compared with placebo |  |  |  |
| The chronic use laxatives may potentiate the effects of diuretics which may lead to significant losses of fluid and electrolytes, including sodium, potassium, magnesium and zinc |  |  |  |
| Concomitant use of large quantities of green tea may increase the effectiveness of anticoagulation drugs (warfarin). |  |  |  |
| Orlistat is contraindicated in patients with cardiovascular diseases |  |  |  |
