# Supplementary material for: Role of community pharmacists in weight management: results of a national study in Lebanon
Source: BMC Health Serv Res. 2020 May 7;20:386. doi: 10.1186/s12913-020-05258-7 (PMC7204056; doi:10.1186/s12913-020-05258-7)
Supplement: Supplementary file 2 — Additional file 2. [file 12913_2020_5258_MOESM2_ESM.rtf]

GET
  FILE='D:\OneDrive - American University of Beirut\Documents\CAM\CAM_HWM\Manuscript\BMC Health Services\R1\SPSS\pharmacy-weight management-02.01.2018 working folder.sav'.
DATASET NAME DataSet1 WINDOW=FRONT.
CORRELATIONS
  /VARIABLES=AGE_regrouped Gender Education_regrouped university_regrouped Received_HWM_training
    WorkExperience_regrouped Queries_WM_day_grpd_2
  /PRINT=TWOTAIL NOSIG
  /MISSING=PAIRWISE.


Correlations


Notes	
Output Created	03-DEC-2019 11:34:02	
Comments		
Input	Data	D:\OneDrive - American University of Beirut\Documents\CAM\CAM_HWM\Manuscript\BMC Health Services\R1\SPSS\pharmacy-weight management-02.01.2018 working folder.sav	
	Active Dataset	DataSet1	
	Filter	<none>	
	Weight	<none>	
	Split File	<none>	
	N of Rows in Working Data File	341	
Missing Value Handling	Definition of Missing	User-defined missing values are treated as missing.	
	Cases Used	Statistics for each pair of variables are based on all the cases with valid data for that pair.	
Syntax	CORRELATIONS
  /VARIABLES=AGE_regrouped Gender Education_regrouped university_regrouped Received_HWM_training
    WorkExperience_regrouped Queries_WM_day_grpd_2
  /PRINT=TWOTAIL NOSIG
  /MISSING=PAIRWISE.	
Resources	Processor Time	00:00:00.03	
	Elapsed Time	00:00:00.04	


[DataSet1] D:\OneDrive - American University of Beirut\Documents\CAM\CAM_HWM\Manuscript\BMC Health Services\R1\SPSS\pharmacy-weight management-02.01.2018 working folder.sav


Correlations	
	AGE_regrouped	Gender of the pharmacist	Education_regrouped	
AGE_regrouped	Pearson Correlation	1	-.165**	-.080	
	Sig. (2-tailed)		.002	.140	
	N	340	340	340	
Gender of the pharmacist	Pearson Correlation	-.165**	1	-.063	
	Sig. (2-tailed)	.002		.247	
	N	340	341	341	
Education_regrouped	Pearson Correlation	-.080	-.063	1	
	Sig. (2-tailed)	.140	.247		
	N	340	341	341	
university_regrouped	Pearson Correlation	-.397**	.313**	.030	
	Sig. (2-tailed)	.000	.000	.588	
	N	338	339	339	
During your university education, did you receive any Home Weight Management (HMW) training?	Pearson Correlation	.125*	.056	-.018	
	Sig. (2-tailed)	.021	.305	.743	
	N	339	340	340	
WorkExperience_regrouped	Pearson Correlation	.759**	-.142**	-.007	
	Sig. (2-tailed)	.000	.009	.904	
	N	339	340	340	
Queries_WM_day_grpd_2	Pearson Correlation	-.022	.013	.009	
	Sig. (2-tailed)	.692	.814	.873	
	N	340	341	341	

Correlations	
	university_regrouped	During your university education, did you receive any Home Weight Management (HMW) training?	WorkExperience_regrouped	
AGE_regrouped	Pearson Correlation	-.397**	.125*	.759**	
	Sig. (2-tailed)	.000	.021	.000	
	N	338	339	339	
Gender of the pharmacist	Pearson Correlation	.313**	.056	-.142**	
	Sig. (2-tailed)	.000	.305	.009	
	N	339	340	340	
Education_regrouped	Pearson Correlation	.030	-.018	-.007	
	Sig. (2-tailed)	.588	.743	.904	
	N	339	340	340	
university_regrouped	Pearson Correlation	1	-.017	-.345**	
	Sig. (2-tailed)		.758	.000	
	N	339	338	338	
During your university education, did you receive any Home Weight Management (HMW) training?	Pearson Correlation	-.017	1	.101	
	Sig. (2-tailed)	.758		.063	
	N	338	340	339	
WorkExperience_regrouped	Pearson Correlation	-.345**	.101	1	
	Sig. (2-tailed)	.000	.063		
	N	338	339	340	
Queries_WM_day_grpd_2	Pearson Correlation	.005	.014	.081	
	Sig. (2-tailed)	.925	.790	.134	
	N	339	340	340	

Correlations	
	Queries_WM_day_grpd_2	
AGE_regrouped	Pearson Correlation	-.022	
	Sig. (2-tailed)	.692	
	N	340	
Gender of the pharmacist	Pearson Correlation	.013	
	Sig. (2-tailed)	.814	
	N	341	
Education_regrouped	Pearson Correlation	.009	
	Sig. (2-tailed)	.873	
	N	341	
university_regrouped	Pearson Correlation	.005	
	Sig. (2-tailed)	.925	
	N	339	
During your university education, did you receive any Home Weight Management (HMW) training?	Pearson Correlation	.014	
	Sig. (2-tailed)	.790	
	N	340	
WorkExperience_regrouped	Pearson Correlation	.081	
	Sig. (2-tailed)	.134	
	N	340	
Queries_WM_day_grpd_2	Pearson Correlation	1	
	Sig. (2-tailed)		
	N	341	

**. Correlation is significant at the 0.01 level (2-tailed).	
*. Correlation is significant at the 0.05 level (2-tailed).	

REGRESSION
  /MISSING LISTWISE
  /STATISTICS COEFF OUTS R ANOVA
  /CRITERIA=PIN(.05) POUT(.10)
  /NOORIGIN
  /DEPENDENT SELF_KNOWLEDGE_SCORE
  /METHOD=ENTER university_regrouped AGE_regrouped_dummy_2 AGE_regrouped_dummy_3 Gender
    Received_HWM_training_switch Queries_WM_day_grpd_2 EducationLevel_3_regrouped_Dummy_2
    EducationLevel_3_regrouped_Dummy_3 WorkExperience_regrouped_dummy_2
    WorkExperience_regrouped_dummy_3.


Regression


Notes	
Output Created	03-DEC-2019 11:45:42	
Comments		
Input	Data	D:\OneDrive - American University of Beirut\Documents\CAM\CAM_HWM\Manuscript\BMC Health Services\R1\SPSS\pharmacy-weight management-02.01.2018 working folder.sav	
	Active Dataset	DataSet1	
	Filter	<none>	
	Weight	<none>	
	Split File	<none>	
	N of Rows in Working Data File	341	
Missing Value Handling	Definition of Missing	User-defined missing values are treated as missing.	
	Cases Used	Statistics are based on cases with no missing values for any variable used.	
Syntax	REGRESSION
  /MISSING LISTWISE
  /STATISTICS COEFF OUTS R ANOVA
  /CRITERIA=PIN(.05) POUT(.10)
  /NOORIGIN
  /DEPENDENT SELF_KNOWLEDGE_SCORE
  /METHOD=ENTER university_regrouped AGE_regrouped_dummy_2 AGE_regrouped_dummy_3 Gender
    Received_HWM_training_switch Queries_WM_day_grpd_2 EducationLevel_3_regrouped_Dummy_2
    EducationLevel_3_regrouped_Dummy_3 WorkExperience_regrouped_dummy_2
    WorkExperience_regrouped_dummy_3.	
Resources	Processor Time	00:00:00.02	
	Elapsed Time	00:00:00.03	
	Memory Required	19008 bytes	
	Additional Memory Required for Residual Plots	0 bytes	


Variables Entered/Removeda	
Model	Variables Entered	Variables Removed	Method	
1	WorkExperience_regrouped=>10 years, Queries_WM_day_grpd_2, AGE_regrouped=31-40, EducationLevel_3_regrouped=Ms/PHD, Received_HWM_training_switch, Gender of the pharmacist, EducationLevel_3_regrouped=pharm D, university_regrouped, WorkExperience_regrouped=4-10 years, AGE_regrouped=>41b	.	Enter	

a. Dependent Variable: SELF_KNOWLEDGE_SCORE	
b. All requested variables entered.	


Model Summary		
Model	R	R Square	Adjusted R Square	Std. Error of the Estimate	Durbin-Watson	
1	.398a	.159	.132	1.437	1.954	

a. Predictors: (Constant), WorkExperience_regrouped=>10 years, Queries_WM_day_grpd_2, AGE_regrouped=31-40, EducationLevel_3_regrouped=Ms/PHD, Received_HWM_training_switch, Gender of the pharmacist, EducationLevel_3_regrouped=pharm D, university_regrouped, WorkExperience_regrouped=4-10 years, AGE_regrouped=>41	


ANOVAa	
Model	Sum of Squares	df	Mean Square	F	Sig.	
1	Regression	122.687	10	12.269	5.939	.000b	
	Residual	650.688	315	2.066			
	Total	773.374	325				

a. Dependent Variable: SELF_KNOWLEDGE_SCORE	
b. Predictors: (Constant), WorkExperience_regrouped=>10 years, Queries_WM_day_grpd_2, AGE_regrouped=31-40, EducationLevel_3_regrouped=Ms/PHD, Received_HWM_training_switch, Gender of the pharmacist, EducationLevel_3_regrouped=pharm D, university_regrouped, WorkExperience_regrouped=4-10 years, AGE_regrouped=>41	


Coefficientsa	
Model	Unstandardized Coefficients	Standardized Coefficients	t	Sig.	
	B	Std. Error	Beta			
1	(Constant)	2.830	.558		5.069	.000	
	university_regrouped	.880	.193	.276	4.554	.000	
	AGE_regrouped=31-40	.050	.250	.016	.201	.841	
	AGE_regrouped=>41	-.095	.335	-.029	-.284	.776	
	Gender of the pharmacist	.172	.173	.056	.998	.319	
	Received_HWM_training_switch	.416	.169	.129	2.468	.014	
	Queries_WM_day_grpd_2	.407	.162	.132	2.520	.012	
	EducationLevel_3_regrouped=pharm D	.126	.203	.034	.619	.536	
	EducationLevel_3_regrouped=Ms/PHD	.791	.212	.209	3.732	.000	
	WorkExperience_regrouped=4-10 years	-.147	.247	-.045	-.596	.551	
	WorkExperience_regrouped=>10 years	-.163	.325	-.053	-.502	.616	

a. Dependent Variable: SELF_KNOWLEDGE_SCORE	

REGRESSION
  /DESCRIPTIVES MEAN STDDEV CORR SIG N
  /MISSING LISTWISE
  /STATISTICS COEFF OUTS R ANOVA COLLIN TOL CHANGE
  /CRITERIA=PIN(.05) POUT(.10)
  /NOORIGIN
  /DEPENDENT SELF_KNOWLEDGE_SCORE
  /METHOD=ENTER university_regrouped AGE_regrouped_dummy_2 AGE_regrouped_dummy_3 Gender
    Received_HWM_training_switch Queries_WM_day_grpd_2 EducationLevel_3_regrouped_Dummy_2
    EducationLevel_3_regrouped_Dummy_3 WorkExperience_regrouped_dummy_2 WorkExperience_regrouped_dummy_3
  /SCATTERPLOT=(*ZRESID ,*ZPRED)
  /RESIDUALS DURBIN HISTOGRAM(ZRESID) NORMPROB(ZRESID)
  /SAVE ZRESID SDBETA.


Regression


Notes	
Output Created	03-DEC-2019 11:54:32	
Comments		
Input	Data	D:\OneDrive - American University of Beirut\Documents\CAM\CAM_HWM\Manuscript\BMC Health Services\R1\SPSS\pharmacy-weight management-02.01.2018 working folder.sav	
	Active Dataset	DataSet1	
	Filter	<none>	
	Weight	<none>	
	Split File	<none>	
	N of Rows in Working Data File	341	
Missing Value Handling	Definition of Missing	User-defined missing values are treated as missing.	
	Cases Used	Statistics are based on cases with no missing values for any variable used.	
Syntax	REGRESSION
  /DESCRIPTIVES MEAN STDDEV CORR SIG N
  /MISSING LISTWISE
  /STATISTICS COEFF OUTS R ANOVA COLLIN TOL CHANGE
  /CRITERIA=PIN(.05) POUT(.10)
  /NOORIGIN
  /DEPENDENT SELF_KNOWLEDGE_SCORE
  /METHOD=ENTER university_regrouped AGE_regrouped_dummy_2 AGE_regrouped_dummy_3 Gender
    Received_HWM_training_switch Queries_WM_day_grpd_2 EducationLevel_3_regrouped_Dummy_2
    EducationLevel_3_regrouped_Dummy_3 WorkExperience_regrouped_dummy_2 WorkExperience_regrouped_dummy_3
  /SCATTERPLOT=(*ZRESID ,*ZPRED)
  /RESIDUALS DURBIN HISTOGRAM(ZRESID) NORMPROB(ZRESID)
  /SAVE ZRESID SDBETA.	
Resources	Processor Time	00:00:01.40	
	Elapsed Time	00:00:01.16	
	Memory Required	19088 bytes	
	Additional Memory Required for Residual Plots	536 bytes	
Variables Created or Modified	ZRE_1	Standardized Residual	
	SDB0_1	Standardized DFBETA for (Constant)	
	SDB1_1	Standardized DFBETA for university_regrouped	
	SDB2_1	Standardized DFBETA for AGE_regrouped_dummy_2	
	SDB3_1	Standardized DFBETA for AGE_regrouped_dummy_3	
	SDB4_1	Standardized DFBETA for Gender	
	SDB5_1	Standardized DFBETA for Received_HWM_training_switch	
	SDB6_1	Standardized DFBETA for Queries_WM_day_grpd_2	
	SDB7_1	Standardized DFBETA for EducationLevel_3_regrouped_Dummy_2	
	SDB8_1	Standardized DFBETA for EducationLevel_3_regrouped_Dummy_3	
	SDB9_1	Standardized DFBETA for WorkExperience_regrouped_dummy_2	
	SDB10_1	Standardized DFBETA for WorkExperience_regrouped_dummy_3	


Descriptive Statistics	
	Mean	Std. Deviation	N	
SELF_KNOWLEDGE_SCORE	5.75	1.543	326	
university_regrouped	1.6288	.48386	326	
AGE_regrouped=31-40	.3436	.47563	326	
AGE_regrouped=>41	.3252	.46915	326	
Gender of the pharmacist	1.49	.501	326	
Received_HWM_training_switch	1.36	.479	326	
Queries_WM_day_grpd_2	1.48	.501	326	
EducationLevel_3_regrouped=pharm D	.22	.413	326	
EducationLevel_3_regrouped=Ms/PHD	.21	.407	326	
WorkExperience_regrouped=4-10 years	.3190	.46681	326	
WorkExperience_regrouped=>10 years	.4448	.49771	326	


Correlations	
	SELF_KNOWLEDGE_SCORE	university_regrouped	AGE_regrouped=31-40	AGE_regrouped=>41	Gender of the pharmacist	Received_HWM_training_switch	Queries_WM_day_grpd_2	
Pearson Correlation	SELF_KNOWLEDGE_SCORE	1.000	.274	.009	-.163	.110	.146	.142	
	university_regrouped	.274	1.000	-.032	-.334	.327	.014	.008	
	AGE_regrouped=31-40	.009	-.032	1.000	-.502	-.159	.069	.009	
	AGE_regrouped=>41	-.163	-.334	-.502	1.000	-.070	-.133	-.018	
	Gender of the pharmacist	.110	.327	-.159	-.070	1.000	-.055	-.013	
	Received_HWM_training_switch	.146	.014	.069	-.133	-.055	1.000	-.028	
	Queries_WM_day_grpd_2	.142	.008	.009	-.018	-.013	-.028	1.000	
	EducationLevel_3_regrouped=pharm D	.023	.159	.010	-.128	.014	-.004	-.036	
	EducationLevel_3_regrouped=Ms/PHD	.138	-.231	-.022	.063	-.160	.060	.076	
	WorkExperience_regrouped=4-10 years	.069	.185	.309	-.391	.048	.055	-.019	
	WorkExperience_regrouped=>10 years	-.146	-.360	-.089	.696	-.143	-.098	.071	
Sig. (1-tailed)	SELF_KNOWLEDGE_SCORE	.	.000	.435	.002	.024	.004	.005	
	university_regrouped	.000	.	.279	.000	.000	.401	.441	
	AGE_regrouped=31-40	.435	.279	.	.000	.002	.106	.434	
	AGE_regrouped=>41	.002	.000	.000	.	.104	.008	.373	
	Gender of the pharmacist	.024	.000	.002	.104	.	.161	.410	
	Received_HWM_training_switch	.004	.401	.106	.008	.161	.	.304	
	Queries_WM_day_grpd_2	.005	.441	.434	.373	.410	.304	.	
	EducationLevel_3_regrouped=pharm D	.337	.002	.432	.010	.401	.471	.259	
	EducationLevel_3_regrouped=Ms/PHD	.006	.000	.348	.130	.002	.140	.085	
	WorkExperience_regrouped=4-10 years	.107	.000	.000	.000	.194	.162	.370	
	WorkExperience_regrouped=>10 years	.004	.000	.055	.000	.005	.039	.101	
N	SELF_KNOWLEDGE_SCORE	326	326	326	326	326	326	326	
	university_regrouped	326	326	326	326	326	326	326	
	AGE_regrouped=31-40	326	326	326	326	326	326	326	
	AGE_regrouped=>41	326	326	326	326	326	326	326	
	Gender of the pharmacist	326	326	326	326	326	326	326	
	Received_HWM_training_switch	326	326	326	326	326	326	326	
	Queries_WM_day_grpd_2	326	326	326	326	326	326	326	
	EducationLevel_3_regrouped=pharm D	326	326	326	326	326	326	326	
	EducationLevel_3_regrouped=Ms/PHD	326	326	326	326	326	326	326	
	WorkExperience_regrouped=4-10 years	326	326	326	326	326	326	326	
	WorkExperience_regrouped=>10 years	326	326	326	326	326	326	326	

Correlations	
	EducationLevel_3_regrouped=pharm D	EducationLevel_3_regrouped=Ms/PHD	WorkExperience_regrouped=4-10 years	WorkExperience_regrouped=>10 years	
Pearson Correlation	SELF_KNOWLEDGE_SCORE	.023	.138	.069	-.146	
	university_regrouped	.159	-.231	.185	-.360	
	AGE_regrouped=31-40	.010	-.022	.309	-.089	
	AGE_regrouped=>41	-.128	.063	-.391	.696	
	Gender of the pharmacist	.014	-.160	.048	-.143	
	Received_HWM_training_switch	-.004	.060	.055	-.098	
	Queries_WM_day_grpd_2	-.036	.076	-.019	.071	
	EducationLevel_3_regrouped=pharm D	1.000	-.271	.069	-.113	
	EducationLevel_3_regrouped=Ms/PHD	-.271	1.000	.021	.072	
	WorkExperience_regrouped=4-10 years	.069	.021	1.000	-.613	
	WorkExperience_regrouped=>10 years	-.113	.072	-.613	1.000	
Sig. (1-tailed)	SELF_KNOWLEDGE_SCORE	.337	.006	.107	.004	
	university_regrouped	.002	.000	.000	.000	
	AGE_regrouped=31-40	.432	.348	.000	.055	
	AGE_regrouped=>41	.010	.130	.000	.000	
	Gender of the pharmacist	.401	.002	.194	.005	
	Received_HWM_training_switch	.471	.140	.162	.039	
	Queries_WM_day_grpd_2	.259	.085	.370	.101	
	EducationLevel_3_regrouped=pharm D	.	.000	.106	.020	
	EducationLevel_3_regrouped=Ms/PHD	.000	.	.352	.097	
	WorkExperience_regrouped=4-10 years	.106	.352	.	.000	
	WorkExperience_regrouped=>10 years	.020	.097	.000	.	
N	SELF_KNOWLEDGE_SCORE	326	326	326	326	
	university_regrouped	326	326	326	326	
	AGE_regrouped=31-40	326	326	326	326	
	AGE_regrouped=>41	326	326	326	326	
	Gender of the pharmacist	326	326	326	326	
	Received_HWM_training_switch	326	326	326	326	
	Queries_WM_day_grpd_2	326	326	326	326	
	EducationLevel_3_regrouped=pharm D	326	326	326	326	
	EducationLevel_3_regrouped=Ms/PHD	326	326	326	326	
	WorkExperience_regrouped=4-10 years	326	326	326	326	
	WorkExperience_regrouped=>10 years	326	326	326	326	


Variables Entered/Removeda	
Model	Variables Entered	Variables Removed	Method	
1	WorkExperience_regrouped=>10 years, Queries_WM_day_grpd_2, AGE_regrouped=31-40, EducationLevel_3_regrouped=Ms/PHD, Received_HWM_training_switch, Gender of the pharmacist, EducationLevel_3_regrouped=pharm D, university_regrouped, WorkExperience_regrouped=4-10 years, AGE_regrouped=>41b	.	Enter	

a. Dependent Variable: SELF_KNOWLEDGE_SCORE	
b. All requested variables entered.	


Model Summaryb	
Model	R	R Square	Adjusted R Square	Std. Error of the Estimate	Change Statistics	
					R Square Change	F Change	df1	
1	.398a	.159	.132	1.437	.159	5.939	10	

Model Summaryb	
Model	Change Statistics	
	df2	Sig. F Change		
1	315	.000	1.954	

a. Predictors: (Constant), WorkExperience_regrouped=>10 years, Queries_WM_day_grpd_2, AGE_regrouped=31-40, EducationLevel_3_regrouped=Ms/PHD, Received_HWM_training_switch, Gender of the pharmacist, EducationLevel_3_regrouped=pharm D, university_regrouped, WorkExperience_regrouped=4-10 years, AGE_regrouped=>41	
b. Dependent Variable: SELF_KNOWLEDGE_SCORE	


ANOVAa	
Model	Sum of Squares	df	Mean Square	F	Sig.	
1	Regression	122.687	10	12.269	5.939	.000b	
	Residual	650.688	315	2.066			
	Total	773.374	325				

a. Dependent Variable: SELF_KNOWLEDGE_SCORE	
b. Predictors: (Constant), WorkExperience_regrouped=>10 years, Queries_WM_day_grpd_2, AGE_regrouped=31-40, EducationLevel_3_regrouped=Ms/PHD, Received_HWM_training_switch, Gender of the pharmacist, EducationLevel_3_regrouped=pharm D, university_regrouped, WorkExperience_regrouped=4-10 years, AGE_regrouped=>41	


Coefficientsa	
Model	Unstandardized Coefficients	Standardized Coefficients	t	Sig.	
	B	Std. Error	Beta			
1	(Constant)	2.830	.558		5.069	.000	
	university_regrouped	.880	.193	.276	4.554	.000	
	AGE_regrouped=31-40	.050	.250	.016	.201	.841	
	AGE_regrouped=>41	-.095	.335	-.029	-.284	.776	
	Gender of the pharmacist	.172	.173	.056	.998	.319	
	Received_HWM_training_switch	.416	.169	.129	2.468	.014	
	Queries_WM_day_grpd_2	.407	.162	.132	2.520	.012	
	EducationLevel_3_regrouped=pharm D	.126	.203	.034	.619	.536	
	EducationLevel_3_regrouped=Ms/PHD	.791	.212	.209	3.732	.000	
	WorkExperience_regrouped=4-10 years	-.147	.247	-.045	-.596	.551	
	WorkExperience_regrouped=>10 years	-.163	.325	-.053	-.502	.616	

Coefficientsa	
Model	Collinearity Statistics	
	Tolerance	VIF	
1	(Constant)			
	university_regrouped	.727	1.376	
	AGE_regrouped=31-40	.448	2.231	
	AGE_regrouped=>41	.258	3.882	
	Gender of the pharmacist	.852	1.174	
	Received_HWM_training_switch	.973	1.028	
	Queries_WM_day_grpd_2	.971	1.029	
	EducationLevel_3_regrouped=pharm D	.899	1.112	
	EducationLevel_3_regrouped=Ms/PHD	.855	1.170	
	WorkExperience_regrouped=4-10 years	.479	2.089	
	WorkExperience_regrouped=>10 years	.243	4.120	

a. Dependent Variable: SELF_KNOWLEDGE_SCORE	


Collinearity Diagnosticsa	
Model	Dimension	Eigenvalue	Condition Index	Variance Proportions	
				(Constant)	university_regrouped	AGE_regrouped=31-40	
1	1	6.622	1.000	.00	.00	.00	
	2	1.536	2.076	.00	.00	.03	
	3	.985	2.593	.00	.00	.01	
	4	.616	3.280	.00	.00	.25	
	5	.530	3.536	.00	.00	.04	
	6	.358	4.301	.00	.01	.05	
	7	.113	7.640	.00	.01	.00	
	8	.099	8.190	.00	.03	.04	
	9	.069	9.776	.00	.00	.49	
	10	.055	10.953	.00	.63	.00	
	11	.017	19.963	.99	.31	.09	

Collinearity Diagnosticsa	
Model	Dimension	Variance Proportions	
		AGE_regrouped=>41	Gender of the pharmacist	Received_HWM_training_switch	Queries_WM_day_grpd_2	EducationLevel_3_regrouped=pharm D	
1	1	.00	.00	.00	.00	.00	
	2	.04	.00	.00	.00	.02	
	3	.00	.00	.00	.00	.31	
	4	.01	.00	.00	.00	.11	
	5	.01	.01	.00	.00	.49	
	6	.13	.02	.01	.01	.03	
	7	.01	.07	.70	.10	.00	
	8	.01	.25	.01	.63	.00	
	9	.68	.00	.02	.04	.01	
	10	.01	.53	.04	.05	.01	
	11	.10	.12	.21	.17	.01	

Collinearity Diagnosticsa	
Model	Dimension	Variance Proportions	
		EducationLevel_3_regrouped=Ms/PHD	WorkExperience_regrouped=4-10 years	WorkExperience_regrouped=>10 years	
1	1	.00	.00	.00	
	2	.01	.05	.02	
	3	.34	.01	.00	
	4	.15	.04	.03	
	5	.39	.06	.01	
	6	.00	.35	.02	
	7	.01	.01	.02	
	8	.05	.01	.00	
	9	.01	.46	.91	
	10	.00	.00	.00	
	11	.04	.00	.00	

a. Dependent Variable: SELF_KNOWLEDGE_SCORE	


Residuals Statisticsa	
	Minimum	Maximum	Mean	Std. Deviation	N	
Predicted Value	4.45	7.22	5.75	.614	326	
Std. Predicted Value	-2.118	2.402	.000	1.000	326	
Standard Error of Predicted Value	.206	.391	.262	.034	326	
Adjusted Predicted Value	4.39	7.28	5.75	.617	326	
Residual	-4.931	3.501	.000	1.415	326	
Std. Residual	-3.431	2.436	.000	.984	326	
Stud. Residual	-3.523	2.463	.000	1.002	326	
Deleted Residual	-5.199	3.580	-.001	1.467	326	
Stud. Deleted Residual	-3.589	2.483	-.001	1.006	326	
Mahal. Distance	5.696	22.998	9.969	2.969	326	
Cook's Distance	.000	.061	.003	.006	326	
Centered Leverage Value	.018	.071	.031	.009	326	

a. Dependent Variable: SELF_KNOWLEDGE_SCORE	


Charts


FREQUENCIES VARIABLES=ZRE_1
  /STATISTICS=RANGE MINIMUM MAXIMUM STDDEV MEAN MEDIAN
  /FORMAT=NOTABLE
  /ORDER=ANALYSIS.


Frequencies


Notes	
Output Created	03-DEC-2019 11:56:01	
Comments		
Input	Data	D:\OneDrive - American University of Beirut\Documents\CAM\CAM_HWM\Manuscript\BMC Health Services\R1\SPSS\pharmacy-weight management-02.01.2018 working folder.sav	
	Active Dataset	DataSet1	
	Filter	<none>	
	Weight	<none>	
	Split File	<none>	
	N of Rows in Working Data File	341	
Missing Value Handling	Definition of Missing	User-defined missing values are treated as missing.	
	Cases Used	Statistics are based on all cases with valid data.	
Syntax	FREQUENCIES VARIABLES=ZRE_1
  /STATISTICS=RANGE MINIMUM MAXIMUM STDDEV MEAN MEDIAN
  /FORMAT=NOTABLE
  /ORDER=ANALYSIS.	
Resources	Processor Time	00:00:00.03	
	Elapsed Time	00:00:00.00	


Statistics	
Standardized Residual  	
N	Valid	326	
	Missing	15	
Mean	.0000000	
Median	.0649548	
Std. Deviation	.98449519	
Range	5.86672	
Minimum	-3.43115	
Maximum	2.43557	

FREQUENCIES VARIABLES=SDB0_1
  /STATISTICS=RANGE MINIMUM MAXIMUM STDDEV MEAN MEDIAN
  /FORMAT=NOTABLE
  /ORDER=ANALYSIS.


Frequencies


Notes	
Output Created	03-DEC-2019 11:57:41	
Comments		
Input	Data	D:\OneDrive - American University of Beirut\Documents\CAM\CAM_HWM\Manuscript\BMC Health Services\R1\SPSS\pharmacy-weight management-02.01.2018 working folder.sav	
	Active Dataset	DataSet1	
	Filter	<none>	
	Weight	<none>	
	Split File	<none>	
	N of Rows in Working Data File	341	
Missing Value Handling	Definition of Missing	User-defined missing values are treated as missing.	
	Cases Used	Statistics are based on all cases with valid data.	
Syntax	FREQUENCIES VARIABLES=SDB0_1
  /STATISTICS=RANGE MINIMUM MAXIMUM STDDEV MEAN MEDIAN
  /FORMAT=NOTABLE
  /ORDER=ANALYSIS.	
Resources	Processor Time	00:00:00.02	
	Elapsed Time	00:00:00.02	


Statistics	
Standardized DFBETA Intercept  	
N	Valid	326	
	Missing	15	
Mean	.0000293	
Median	.0000816	
Std. Deviation	.05949995	
Range	.57300	
Minimum	-.27381	
Maximum	.29919	

FREQUENCIES VARIABLES=SDB2_1 SDB3_1 SDB4_1 SDB5_1 SDB6_1 SDB7_1 SDB8_1 SDB9_1 SDB10_1
  /STATISTICS=RANGE MINIMUM MAXIMUM STDDEV MEAN MEDIAN
  /FORMAT=NOTABLE
  /ORDER=ANALYSIS.


Frequencies


Notes	
Output Created	03-DEC-2019 11:58:07	
Comments		
Input	Data	D:\OneDrive - American University of Beirut\Documents\CAM\CAM_HWM\Manuscript\BMC Health Services\R1\SPSS\pharmacy-weight management-02.01.2018 working folder.sav	
	Active Dataset	DataSet1	
	Filter	<none>	
	Weight	<none>	
	Split File	<none>	
	N of Rows in Working Data File	341	
Missing Value Handling	Definition of Missing	User-defined missing values are treated as missing.	
	Cases Used	Statistics are based on all cases with valid data.	
Syntax	FREQUENCIES VARIABLES=SDB2_1 SDB3_1 SDB4_1 SDB5_1 SDB6_1 SDB7_1 SDB8_1 SDB9_1 SDB10_1
  /STATISTICS=RANGE MINIMUM MAXIMUM STDDEV MEAN MEDIAN
  /FORMAT=NOTABLE
  /ORDER=ANALYSIS.	
Resources	Processor Time	00:00:00.03	
	Elapsed Time	00:00:00.03	


Statistics	
	Standardized DFBETA AGE_regrouped_dummy_2	Standardized DFBETA AGE_regrouped_dummy_3	Standardized DFBETA Gender	Standardized DFBETA Received_HWM_training_switch	Standardized DFBETA Queries_WM_day_grpd_2	
N	Valid	326	326	326	326	326	
	Missing	15	15	15	15	15	
Mean	.0000274	-.0000533	.0000226	-.0000674	.0000008	
Median	.0001711	.0001065	.0000962	.0000880	-.0008956	
Std. Deviation	.05846003	.06758370	.05641902	.05811845	.05548680	
Range	.59674	.98107	.42163	.50831	.36604	
Minimum	-.21926	-.56704	-.18919	-.32665	-.20289	
Maximum	.37749	.41403	.23244	.18166	.16316	

Statistics	
	Standardized DFBETA EducationLevel_3_regrouped_Dummy_2	Standardized DFBETA EducationLevel_3_regrouped_Dummy_3	Standardized DFBETA WorkExperience_regrouped_dummy_2	Standardized DFBETA WorkExperience_regrouped_dummy_3	
N	Valid	326	326	326	326	
	Missing	15	15	15	15	
Mean	.0000094	.0000355	-.0000307	.0000116	
Median	-.0002101	-.0000144	.0000018	.0000141	
Std. Deviation	.06126481	.05256327	.05585250	.06084808	
Range	.58179	.42781	.54989	.79194	
Minimum	-.36856	-.21140	-.30522	-.43883	
Maximum	.21323	.21641	.24467	.35311	
